# Our Publisher

**Published:** 1834

**Authors:** 


### II.—ORIGINAL.

### The Western Journal.

*E Sylvis Nuncius.*

CINCINNATI, JULY 1, 1834.

### I.—OUR PUBLISHER.

Our Publisher, ever desirous of promoting the interests of his subscribers, has resolved on a measure, that will reduce the amount of postage on the Journal to one half. It is simply to print it on paper double the size of that heretofore used; so that, but half the number of sheets will be necessary, and consequently but half the amount of postage be chargeable. Many postmasters may not be fully aware of this change, until it is explained to them by our subscribers. The difficulty of obtaining enlarged paper has led to some loss of time in the issuing of this number, but the same cause of delay will not exist in future.

